# The implementation of e-learning tools to enhance undergraduate bioinformatics teaching and learning: a case study in the National University of Singapore

**DOI:** 10.1186/1471-2105-10-S15-S12

**Published:** 2009-12-03

**Authors:** Shen Jean Lim, Asif Mohammad Khan, Mark De Silva, Kuan Siong Lim, Yongli Hu, Chay Hoon Tan, Tin Wee Tan

**Affiliations:** 1 Department of Biochemistry, Yong Loo Lin School of Medicine, National University of Singapore, 8 Medical Drive, Singapore 117597; 2 Life Sciences Institute, National University of Singapore, Centre for Life Sciences, 28 Medical Drive, Singapore 117456; 3 Department of Biological Sciences, National University of Singapore, 14 Science Drive 4, Singapore 117543; 4 Department of Pharmacology, Yong Loo Lin School of Medicine, National University of Singapore, 10 Medical Drive, Singapore 117597

## Abstract

**Background:**

The rapid advancement of computer and information technology in recent years has resulted in the rise of e-learning technologies to enhance and complement traditional classroom teaching in many fields, including bioinformatics. This paper records the experience of implementing e-learning technology to support problem-based learning (PBL) in the teaching of two undergraduate bioinformatics classes in the National University of Singapore.

**Results:**

Survey results further established the efficiency and suitability of e-learning tools to supplement PBL in bioinformatics education. 63.16% of year three bioinformatics students showed a positive response regarding the usefulness of the Learning Activity Management System (LAMS) e-learning tool in guiding the learning and discussion process involved in PBL and in enhancing the learning experience by breaking down PBL activities into a sequential workflow. On the other hand, 89.81% of year two bioinformatics students indicated that their revision process was positively impacted with the use of LAMS for guiding the learning process, while 60.19% agreed that the breakdown of activities into a sequential step-by-step workflow by LAMS enhances the learning experience

**Conclusion:**

We show that e-learning tools are useful for supplementing PBL in bioinformatics education. The results suggest that it is feasible to develop and adopt e-learning tools to supplement a variety of instructional strategies in the future.

## Background

Recent years have witnessed the emergence of bioinformatics scientists as an increasingly important discipline of biology - the marriage between biology and computer science, for the managing and mining of biological data to complement, speed up and expand the practice of biological research. The advent of bioinformatics has created a growing demand in the research industry for well-trained graduates specializing in the discipline to be met by education institutions through effective incorporation and teaching of bioinformatics in the traditional life sciences curriculum [1].

In view of the high demand for bioinformatics scientist, many universities in various countries today have already incorporated bioinformatics, in part or in whole, in their undergraduate and graduate courses [2-5]. In the context of bioinformatics education, due to the multi-disciplinary and computer-intensive nature of bioinformatics, conventional pedagogy methods for biology education have to be revised and novel pedagogy strategies have to be adopted and customized to better suit the teaching and learning needs of the field [6].

With the rapid advancement of computer and information technology in the modern age, e-learning technologies, such as Learning Activity Management System (LAMS) and wiki, have been implemented to enhance and complement traditional classroom teaching, promising more efficient and hassle-free alternatives for the preparation of teaching materials, evaluation of students' progress and performance and management of classroom data and statistics [6,7]. For the learners, on the other hand, e-learning offers them a greater degree of control over their learning pace and experience. In a discipline such as bioinformatics, which relies heavily on the use of computers, e-learning is particularly advantageous and convenient for the delivery of instant, hands-on learning experience.

Due to its emphasis on independent, self-directed learning and the shift in the focus of the learning experience from the teachers to the students, e-learning is particularly suited for problem-based learning (PBL) as well. PBL, first introduced by McMaster University medical school in 1969, is an innovative collaborative teaching/learning instructional strategy in which students collaboratively solve problems under the guidance and support of facilitators [8,9]. In general, a typical PBL learning cycle involves the introduction of a problem scenario, small group discussions with an appointed leader and scribe, the presentation of a potential solution and evaluation. The learning process is known to promote active participation, problem-solving and self-directed learning, thus transforming the traditional learning experience from teacher-directed instruction to student-centered learning and offering a new source of empowerment for the students [10].

In this paper, we recount the experience of implementing e-learning technology to complement the PBL strategy adopted in the teaching of two undergraduate bioinformatics classes (year two and year three) that are part of the life sciences course offered in the National University of Singapore.

## Results and discussion

**Figure 1:**
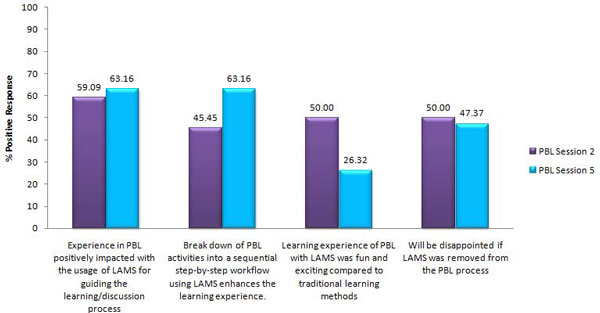
**Graphical comparison of the percentage of positive responses by the year three bioinformatics cohort on four survey questions regarding the use of LAMS after PBL Sessions 2 and 5**. For each question, students' responses were considered to be positive when any one of the following options was checked: slightly enhanced, greatly enhanced, agree or strongly agree.  Negative responses included the selection of the options neutral, disagree or strongly disagree.

A variety of software to support PBL was introduced to a year three and a year two bioinformatics and biocomputing course, comprising of 26 and 252 undergraduate students, respectively. The Learning Activity Management System (LAMS) [11], developed by the Macquarie E-Learning Centre for Excellence (MELCOE), Australia was used for the asynchronous delivery of teaching/learning content. LAMS is a revolutionary new tool for managing and delivering online collaborative learning activities that can be linked together to form an intuitive learning sequence (for an example, see additional file 1: S.Figure1). With LAMS, instructors are able to monitor the progress of students real-time, allowing for efficient and timely assessment and feedback. Students, on the other hand, are free to execute the learning sequence at their own pace and interact with others as well as the instructors. The wiki technology was also implemented in the course to foster collaborative authoring of progress and learning reports. A key advantage of the wiki technology lies in its gentle learning curve and the ease of which pages can be created and edited [12,13].

### Implementation of problem-based learning and e-learning tools in year three bioinformatics education

The efficiency and suitability of e-learning tools in complementing PBL in bioinformatics education was evaluated based on surveys integrated in the PBL sessions. Based on the results of the survey, the class of 26 students generally recognized the usefulness of LAMS in guiding the learning and discussion process involved in problem-based learning and in enhancing the learning experience by breaking down PBL activities into a sequential workflow (Figure 1). For the former aspect, there is an increase in the percentage of positive response by students from 59.09% to 63.16% over the PBL sessions whereas for the latter, there was an increase in the percentage of positive response from 45.45% to 63.16% over the PBL sessions. These results serve to establish the efficiency and suitability of LAMS as a collaborative teaching/learning tool to supplement PBL.

Nevertheless, though LAMS was appreciated by the year three bioinformatics students, it was not regarded as a fun and exciting way of learning compared to traditional learning methods. This is evident from the drop in the relevant percentage positive response from 50% to 26.32% over the five PBL sessions. In addition, there was a decrease in the number of students (from 50% to 47.37%) indicating that they will be disappointed if LAMS was to be removed from the PBL process. Although this may be attributed to the increasing workload and difficulty of the PBL material delivered over the PBL sessions, it highlights the necessity of developing more creative and flexible LAMS sequences to incorporate more elements of fun so as to stimulate greater interest in learning among students.

**Figure 2:**
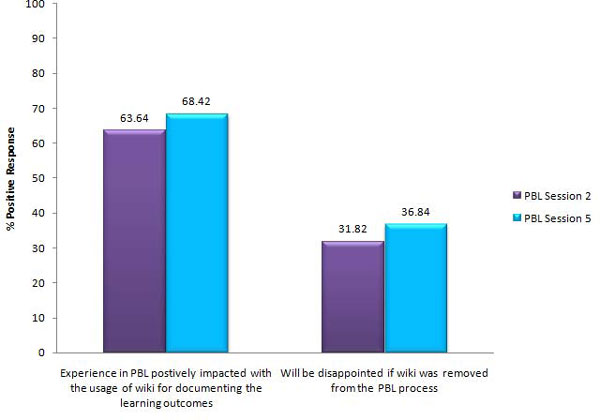
**Graphical comparison of the percentage of positive responses by the year three bioinformatics cohort on two survey questions on the use of wiki after PBL Sessions 2 and 5**. For each question, students' responses were considered to be positive when any one of the following options was checked by stuedents in the feedback: slightly enhanced, greatly enhanced, agree or strongly agree.  Negative responses included the selection of the options neutral, disagree or strongly disagree.

In terms of the effectiveness of wiki for year three bioinformatics teaching, more than half of the class agreed that their experience in PBL was enhanced positively through the use of wiki for documenting the PBL process, coordinating the assignments and compiling the learning issues, hypothesis and conclusions, with an observed increase in the percentage positive response from 63.64% to 68.42% over the PBL sessions (Figure 2). There was also an increase in the percentage positive response from students who indicated disappointment if wiki was to be removed from the PBL sessions; however, this was only indicated by less than half of the class (31.82% and 36.84% respectively for Sessions 2 and 5), with most of the students (36.36% and 36.84% respectively) taking a neutral stand (data not shown).

### Implementation of e-learning tools in year two bioinformatics education

**Figure 3:**
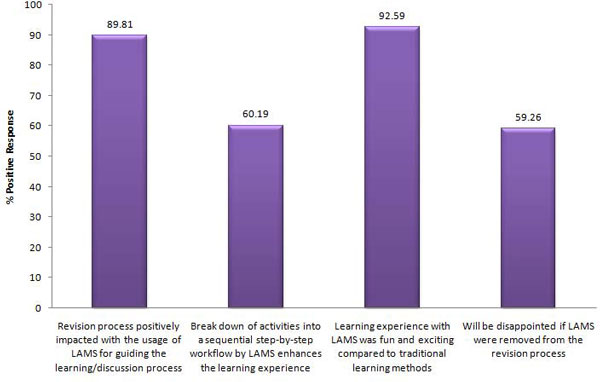
**The percentage of positive responses by the year two bioinformatics cohort on four survey questions regarding the use of LAMS**. For each question, students' responses were considered to be positive when any one of the following options was checked: slightly enhanced, greatly enhanced, agree or strongly agree.  Negative responses included the selection of the options neutral, disagree or strongly disagree.

Compared to the year three bioinformatics students, higher percentages of positive responses were generally observed from the students taking the year two bioinformatics course (Figure 3).

According to the survey results, 89.81% of the students agreed that their revision process was positively impacted with the use of LAMS for guiding the learning process, whereas 60.19% of the students thought that the breakdown of activities into a sequential step-by-step workflow using LAMS enhanced the learning experience. Feedback through the open-response questions showed positive comments from the year two bioinformatics students expressing their wish for LAMS to be integrated in the course throughout the semester as a tutorial and revision tool.

In contrast to the LAMS survey results from the year three class, 92.59% (Figure 3) of the students from the year two bioinformatics class found learning with LAMS more fun and exciting than traditional learning methods while 59.26% of the students indicated disappointment if LAMS was to be removed from the revision process. Nevertheless, there were some suggestions by the students that LAMS can be made more interactive.

### Challenges

Based on the general feedback on LAMS by the year two and year three bioinformatics cohort, it is evident that more elements of interactivity, fun and excitement need to be introduced into LAMS to capture the students' interest and increase their motivation in self-learning. Though it is possible that the lower percentage of positive responses observed among the year three bioinformatics students regarding the fun and excitement of using LAMS may be due to the increased frustration and fatigue from the higher amount of workload compared to the year two students.  This highlights an inevitable need for further improvement and fine tune of the existing learning sequences to increase their interactivity without compromising on the workload.

On the other hand, due to its emphasis on collaborative efforts and group interactions, PBL, in general, appears to be more appropriate for small group teaching. Therefore, in a realistic view, the implementation of PBL and LAMS is more feasible for classes of smaller size (comprising of 10-15 students), whereas resources, such as facilitators and computers, are not strained. This was clear from the challenge encountered in this prokect in implementing PBL and LAMS in the large class size of the year two bioinformatics cohort (252 students), especially when synchronous teaching is involved. In this context, the breaking down of a large class into small PBL discussion groups poses difficulties due to limitations in resources. For such classes, the implementation of PBL is not feasible and the utility of LAMS is limited to basic functionality. In view of these limitations, more effective ways of implementing PBL and LAMS in large classes need to be explored. A possible future direction is to explore the adoption of other novel teaching and learning strategies that are more applicable to large class size.

## Conclusion

It is shown herein that e-learning tools such as LAMS and wiki are promising for supplementing PBL in bioinformatics education. The main benefit of such innovative teaching strategy over traditional teaching methods lies in the promotion of independent and collaborative learning/teaching through group discussions with minimal guidance from the facilitator. In this respect, e-learning tools are useful in complementing PBL by providing the necessary resources and support for the tasks involved in learning, as well as teaching and monitoring. Though certain limitations and challenges are present, taken together, the feedback and response obtained from the bioinformatics students in the National University of Singapore are encouraging and provides impetus for the further development and implementation of such e-learning technology in bioinformatics education.

## Methods

### LAMS server hosting

One-year contract LAMS server hosting service was purchased from the LAMS foundation[11]. The server is a shared server with 2 GB of disk space which can support a maximum number of 200 users at once and a maximum bandwidth of 5 GB per month. It was installed with LAMS (version 2.2) and can be accessed at http://nus.lamsinternational.com.

To ensure that LAMS remains available after the contract hosting service, pre-paid credit was purchased from GoGrid[14] for the testing and customization of LAMS on the cloud computing server. The cloud computing server (a 32-bit Windows 2008 server) is installed with the latest version of LAMS (version 2.3) and can be accessed at http://biocloud.bic.nus.edu.sg:8080/lams.

### Wiki server hosting

MediaWiki 1.5.5. [15], a free software wiki package written in PHP, was installed in a server with Intel Pentium 4 3.0 GHz CPU and 1 GB of RAM. The wiki site is residing on a hard disk of approximately 80 GB that has been reserved for web hosting.

### Implementation of problem-based learning and e-learning tools in year three bioinformatics education

A typical PBL session in the year three bioinformatics class involved the introduction of a problem scenario, which the students were required to solve collaboratively under the guidance of their facilitators. LAMS was used as a teaching tool to present the problem scenario, define issues to be addressed, assess the students' understanding of the problem scenario through a series of questions and answers (Q&As) and finally, to provide constructive feedback and critique to the facilitator and group members (See Additional file 1: Figure S1). A discussion forum was also created in LAMS to facilitate students' discussion and exchange of ideas. After each PBL session, students were required to record their progress and findings on a wiki site maintained by an in-house server and to prepare a presentation for the whole class.

Towards the end of the course, LAMS was used as an optional e-revision tool for the students to help them prepare for the examinations (See Additional file 2: Figure S2). The LAMS sequence was designed as a series of multiple choice questions (MCQs) organized under different sections corresponding to each section of the curriculum.

Surveys were integrated in LAMS during the second and last (fifth) PBL sessions in order to collect the students' opinion on PBL and LAMS to facilitate the refinement and improvement in the design of the PBL sessions as well as the LAMS sequences.

### Implementation of e-learning tools in year two bioinformatics education

LAMS was used as an optional e-revision tool, designed as a series of multiple choice questions (MCQs) pertaining to different parts of the curriculum. A survey was added into the LAMS revision sequence to solicit relevant feedback.

## Competing interests

The authors declare that they have no competing interests.

## Authors' contributions

TTW designed the study, while all authors executed the project and analyzed the findings. All authors have read and approved of this manuscript.

## Note

Other papers from the meeting have been published as part of *BMC Genomics *Volume 10 Supplement 3, 2009: Eighth International Conference on Bioinformatics (InCoB2009): Computational Biology, available online at http://www.biomedcentral.com/1471-2164/10?issue=S3.

## Supplementary Material

Additional file 1 **Figure S1: An example of a LAMS sequence implemented during a typical PBL session**. The LAMS sequence included the use of a noticeboard tool to present the problem scenario, followed by a task list tool to define the list of skills to be acquired through the PBL session, a Q&A tool to assess the students' understanding of the problem scenario, a task list tool to define the minimum results required for presentation and a survey tool for students to provide feedback and critique to their group members. Click here for file

Additional file 2 **Figure S2: An example of a LAMS sequence implemented as a revision tool**. For each topic of the LSM2104/LSM3241 curriculum, a multiple choice tool was used to create revision questions. These questions were preceded by a notice board tool to provide instructions to the students and were followed by a survey tool to collect feedback from them. Click here for file
