# Supplementary material for: The implementation of e-learning tools to enhance undergraduate bioinformatics teaching and learning: a case study in the National University of Singapore
Source: BMC Bioinformatics. 2009 Dec 3;10(Suppl 15):S12. doi: 10.1186/1471-2105-10-S15-S12 (PMC2788352; doi:10.1186/1471-2105-10-S15-S12)

The implementation of e-learning tools to enhance undergraduate bioinformatics teaching and learning: a case study in the National University of Singapore

by

Shen Jean Lim, Asif Mohammad Khan, Mark De Silva, Kuan Siong Lim, Yongli Hu, Chay Hoon Tan and Tin Wee Tan

Additional File 1:

Figure S1: An example of a LAMS sequence implemented during a typical PBL session
The LAMS sequence includes the use of a noticeboard tool to present the problem scenario, followed by a task list tool to define the list of skills to be acquired through the PBL session, a Q&A tool to assess the students' understanding of the problem scenario, a task list tool to define the minimum results required for presentation and a survey tool for students to provide feedback and critique to their group members.

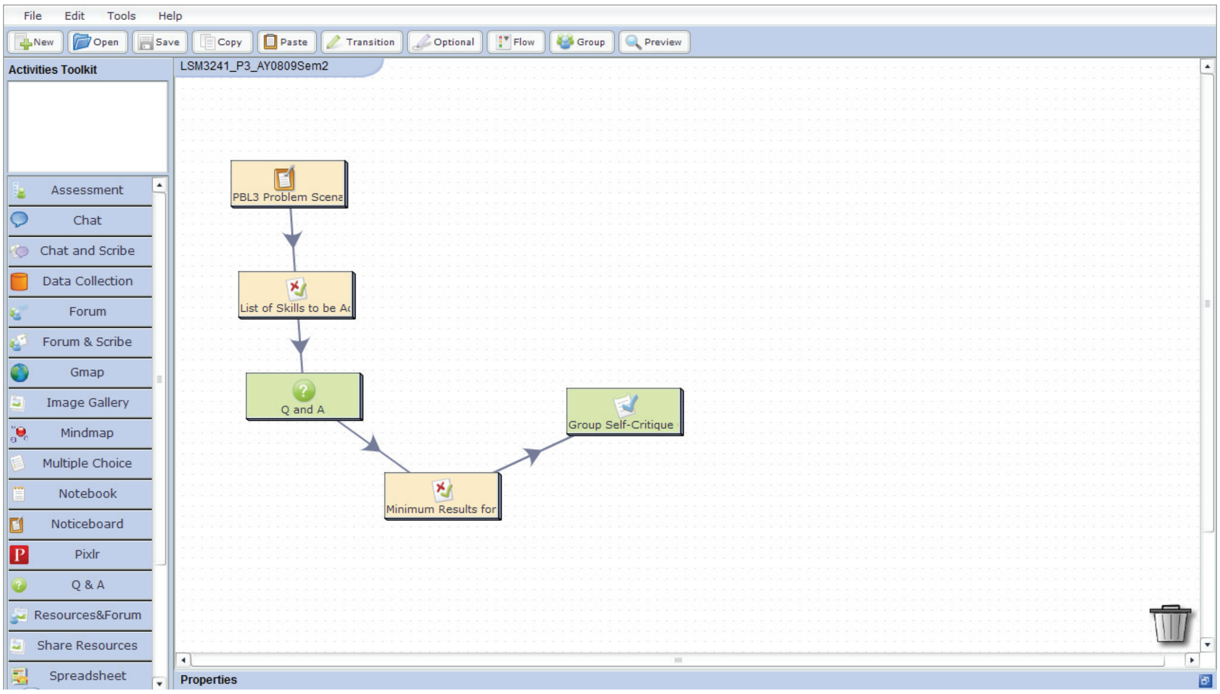

Supplement: Additional file 1 — Figure S1: An example of a LAMS sequence implemented during a typical PBL session. The LAMS sequence included the use of a noticeboard tool to present the problem scenario, followed by a task list tool to define the list of skills to be acquired through the PBL session, a Q&A tool to assess the students' understanding of the problem scenario, a task list tool to define the minimum results required for presentation and a survey tool for students to provide feedback and critique to their group members. [file 1471-2105-10-S15-S12-S1.pdf]
