# Supplementary material for: The implementation of e-learning tools to enhance undergraduate bioinformatics teaching and learning: a case study in the National University of Singapore
Source: BMC Bioinformatics. 2009 Dec 3;10(Suppl 15):S12. doi: 10.1186/1471-2105-10-S15-S12 (PMC2788352; doi:10.1186/1471-2105-10-S15-S12)

The implementation of e-learning tools to enhance undergraduate bioinformatics teaching and learning: a case study in the National University of Singapore

by

Shen Jean Lim, Asif Mohammad Khan, Mark De Silva, Kuan Siong Lim, Yongli Hu, Chay Hoon Tan and Tin Wee Tan

Additional File 2:

Figure S2: An example of a LAMS sequence implemented as a revision tool

For each section of the LSM2104/LSM3241 curriculum, a multiple choice tool is used to create revision questions. The revision questions are preceded by a notice board tool to provide instructions to the students and followed by a survey tool to collect feedback from students..

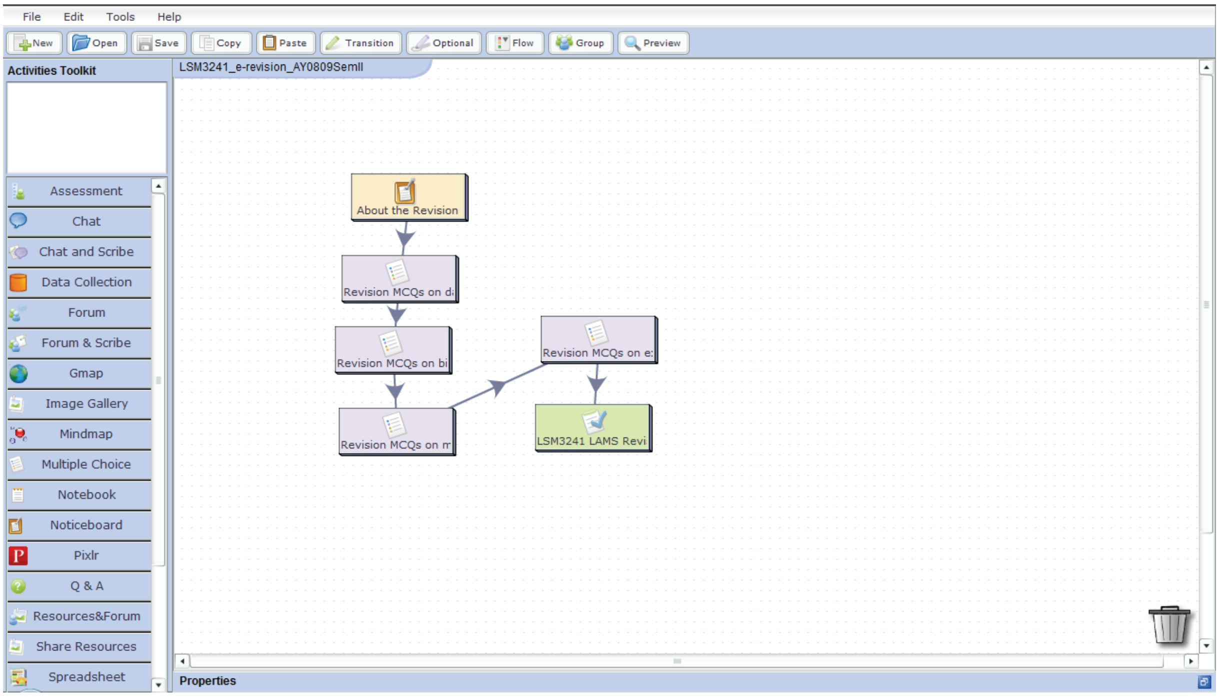

Supplement: Additional file 2 — Figure S2: An example of a LAMS sequence implemented as a revision tool. For each topic of the LSM2104/LSM3241 curriculum, a multiple choice tool was used to create revision questions. These questions were preceded by a notice board tool to provide instructions to the students and were followed by a survey tool to collect feedback from them. [file 1471-2105-10-S15-S12-S2.pdf]
